# Supplementary material for: D-alanine aminotransferase (Dat) promotes Staphylococcus aureus colonization fitness on human nasal respiratory epithelium
Source: bioRxiv. 2026 Jun 2:2026.06.01.729472. Preprint. [Version 1] doi: 10.64898/2026.06.01.729472 (PMC13251936; doi:10.64898/2026.06.01.729472)
Supplement: Supplement 1 [file NIHPP2026.06.01.729472v1-supplement-1.pdf]

bioRxiv preprint doi: https://doi.org/10.64898/2026.06.01.729472; this version posted June 4, 2026. The copyright holder for this preprint (which was not certified by peer review) is the author/funder, who has granted bioRxiv a license to display the preprint in perpetuity. It is made available under a CC-BY 4.0 International license.

Supporting Information.

bioRxiv preprint doi: https://doi.org/10.64898/2026.06.01.729472; this version posted June 4, 2026. The copyright holder for this preprint (which was not certified by peer review) is the author/funder, who has granted bioRxiv a license to display the preprint in perpetuity. It is made available under a CC-BY 4.0 International license.

Figure S1. HNO-ALI cytotoxicity under different colonization conditions

Figure S2. P_{dat} promoter prediction and orientation of P_{dat}*dat* on chromosome

Figure S3. Parent vs. mutant strain *in vitro* growth curves

Figure S4. CFUs/HNO-ALI competing *dat*::Tn vs. parent in other clonal complexes

Table S1. TnSeq analysis

Table S2. Statistical analyses

Table S3. P_{dat} region promoter prediction results

Table S4. Bacterial strains and plasmids

Table S5. Oligonucleotide primers

Supporting Materials.

D-alanine aminotransferase (Dat) promotes *Staphylococcus aureus* colonization fitness on human nasal respiratory epithelium

Figure S1. HNO-ALI cytotoxicity under different colonization conditions

Figure S2. P_{dat} promoter prediction and orientation of P_{dat}*dat* on chromosome

Figure S3. Parent vs. mutant strain *in vitro* growth curves

Figure S4. CFUs/HNO-ALI competing *dat*::Tn vs. parent in other clonal complexes

Table S1. TnSeq analysis (as a separate xlsx file)

- **S1A.** TnSeq Gene Level Analysis
- **S1B.** STRING Enrichment Analysis

Table S2. Statistical analyses (as a separate xlsx file)

- **S2A.** HNO-ALI CFU Competition Analysis
- **S2B.** Growth Curves OD600
- **S2C.** HNO-ALI CFU Complementation Analysis
- **S2D.** *S. aureus* coculture with *C. pseudodiphtheriticum*
- **S2E.** Cytotoxicity - LDH

Table S3. P_{dat} region promoter prediction results

Table S4. Bacterial strains and plasmids

Table S5. Oligonucleotide primers

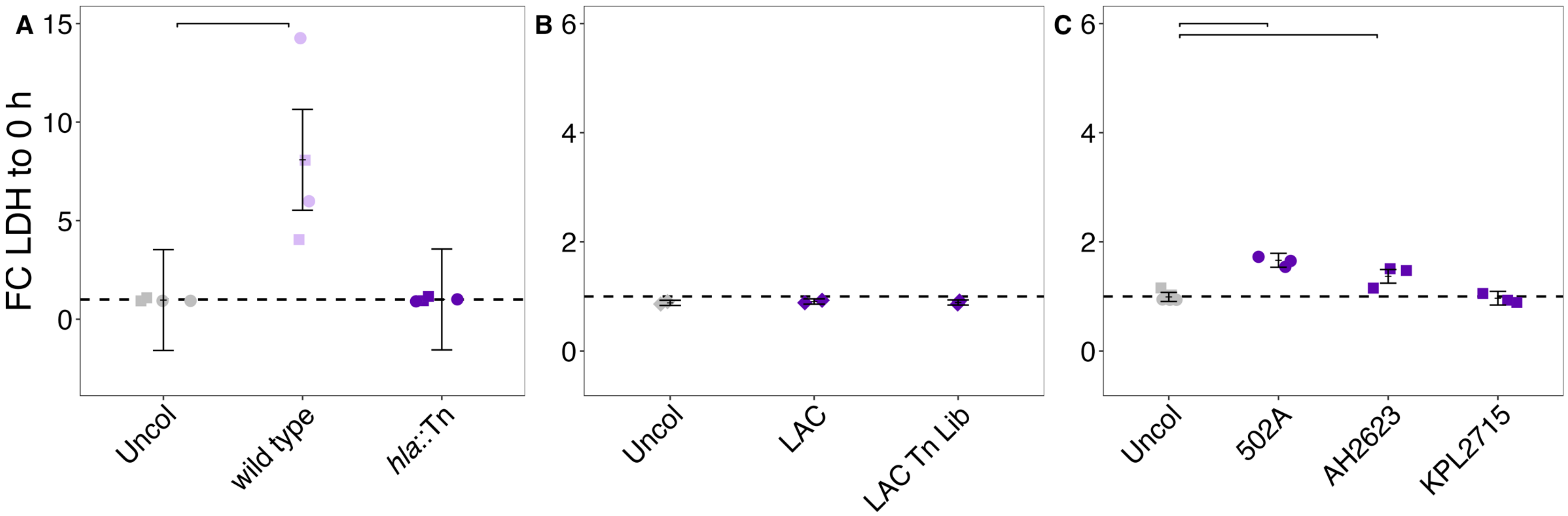

Figure S1. Cytotoxicity profiling of *S. aureus* strains on HNO–ALI. (**A**) *S. aureus* LAC induced cytotoxicity by 24 h in an *hla*-dependent manner in HNO-ALI generated using a recently revised protocol that has improved on-time production. (**B**) No cytotoxicity was caused by LAC (CC8) or LAC Tn library after 8 h colonization in current HNO-ALI. (**C**) There was minimal HNO-ALI cytotoxicity caused by 502A (CC30) and AH2623 (CC5) by 16 h of colonization and no apparent toxicity caused by KPL2715 (CC30) after 24 h colonization. LDH was quantified as a fold change from the final timepoint of colonization compared to the amount of LDH detected at 1 hour before colonization. $n \geq 2$ independent experiments in a minimum of 1 HNO line (HNO204, circles; HNO918, squares; or HNO919, diamonds). Fold change between groups was compared using an LMM with colonizing strain as fixed effect and experimental date as a random effect, with contrasts performed relative to the uncolonized condition and adjusted for multiple comparisons using the Holm method. Horizontal brackets represent statistically significant comparisons $p < 0.05$. Vertical brackets represent the model-predicted mean values and confidence intervals ± 2 x SEM. Statistical analysis is in **Table S2E**.

A

5′ TAGGACATTTATTAGTGTTTCAGGTTCTTTATCGTTAGTCATAGCTAATATGCATATAGAAAGAAGAATACATGCGTGAAACTCATTCATAAGAAATACTAATATC
TAAAGAAAACGTATTTCTTTATCTTTTCGAAATTCTCTGTGTTGGGGCCCTTACCCCAACTTGCACATTATTGAAAGCTGACTATTGGCCAGCTTCTGTGTTGGGGCC
CTTACCCCAACTTGCACATTATTGAAAGCTAACTATTGGCCAGCTTCTATGTTGGGGCCCCGCCAACTTGCATTGTCTGTAGAATTTCTTTTCGAAATTCTCTGTGTT
GGGGCCCCGGGGCGCATTTTCGTTCGGTTAATTACTGTCAATATTAAATTGTGTGCCTAGTACATTGACTTATATCTCGAGCTCTTGAATATGTGTATTTTAAATATA
TAAATTAAAAAAGGAACAAAGCAGCTATTTTATGATTTGTGTCATATCAATAGCATCGTTTTGTTCCTTTTTATTATTTTCTGACATCTTAT**TGTAGTTTTTATATCTT**
TTCATGTAAAATAGAATTTAACTTATAGAAAAAAGCAACTTCGATTTCGA

bioRxiv preprint doi: https://doi.org/10.64898/2026.06.01.729472; this version posted June 4, 2026. The copyright holder for this preprint (which was not certified by peer review) is the author/funder, who has granted bioRxiv a license to display the preprint in perpetuity. It is made available under a CC-BY 4.0 International license.

>*pepV* (SAUSA300_RS09270)

ATGTGGAAAGAAAAAGTTCAACAATACGAAGATCAAATCATTAATGACTTAAAAGGATTATTAGCAATTGAAAGTGTGAGAGATGATGCAAAAGCATCAGAAGACGCA
CCAGTTGGTCCAGGTCCTCGTAAGGCATTAGACTACATGTATGAAATTGCACATAGAGATGGATTTACAACACATGATGTGGATCATATTGCAGGAAGAATTGAGGCA
GGTAAAGGAAATGACGTATTAGGTATCTTATGTCATGTTGACGTTGTTCCTGCTGGTGATGGATGGGATAGTAATCCGTTCGAGCCGGTTGTAACAGAAGATGCTATC
ATAGCTAGAGGTACACTTGATGACAAAGGTCCAACAATTGCTGCTTATTATGCAATTAAGATATTAGAAGATATGAATGTGGATTGGAAGAAACGTATTCATATGATT
ATTGGTACGGATGAAGAATCTGATTGGAAATGTACGGATCGCTATTTTAAAACAGAAGAAATGCCAACATTAGGTTTTGCACCAGATGCAGAATTTCCATGTATTCAT
GGTGAAAAAGGCATTACAACATTTGATTTAGTTCAAAATAAACTTACTGAAGATCAAGATGAACCTGATTATGAATTAATAACTTTTAAATCTGGTGAACGTTACAAC
ATGGTACCTGATCATGCAGAAGCAAGAGTGCTTGTTAAAGAAAATATGACAGATGTTATTCAAGACTTTGAGTACTTTTTAGAACAAAATCATTTACAAGGTGATAGT
ACTGTTGATAGTGGCATTCTAGTTTTAACAGTTGAAGGTAAAGCGGTTCATGGTATGGATCCATCTATCGGTGTGAATGCGGGTCTTTACTTACTAAAATTCTTAGCA
TCATTAAATCTTGATAATAATGCACAAGCGTTTGTAGCATTTAGTAATCGCTACTTATTTAATTCAGATTTTGGTGAAAAGATGGGAATGAAATTCCATACAGATGTC
ATGGGTGACGTGACAACTAACATTGGTGTTATTACATATGATAATGAAAACGCAGGTCTTTTCGGTATCAACTTACGCTACCCAGAAGGATTTGAATTTGAAAAAGCT
ATGGATCGTTTTGCAAATGAGATTCAACAATATGGCTTTGAAGTGAAATTAGGTAAAGTCCAACCACCACATTATGTTGATAAAAATGATCCTTTTGTACAAAAGTTA
GTTACTGCATATAGAAATCAAACAAATGATATGACTGAACCTTATACTATAGGTGGCGGTACTTATGCGAGAAACTTAGACAAGGGTGTAGCATTTGGCGCAATGTTT
AGTGATTCTGAAGATTTAATGCATCAGAAAAATGAATATATCACTAAAAAACAGTTATTTAACGCAACTAGTATTTACTTAGAAGCAATTTATTCATTATGCGTGGAG
GAA**TAA**TAT **3′**

>*dat* (SAUSA300_RS09265)

ATG

B

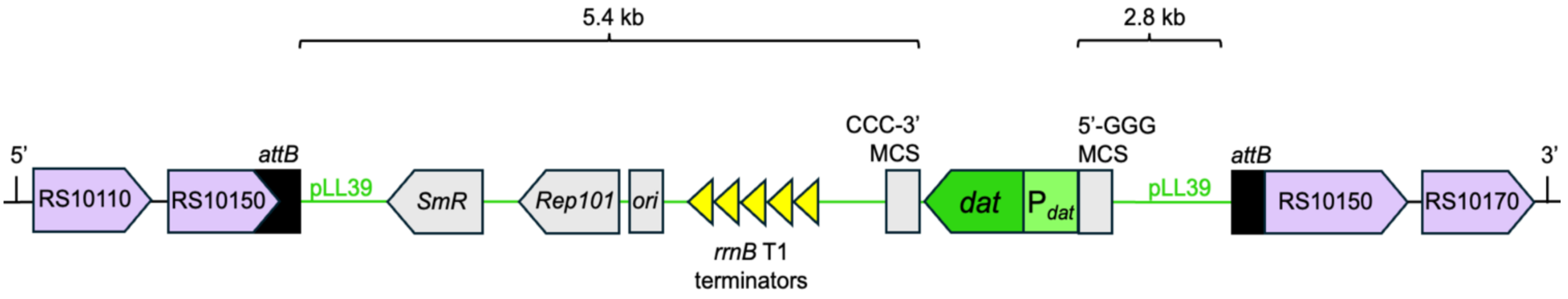

Figure S2. P$_{dat}$ is the only promoter likely to promote *dat* transcription in KPL4549 (*dat*::Tn P$_{dat}$*dat*). (A) SAPPHIRE-predicted promoter sequences identified 5′ of and within the *pepV* coding region of *S. aureus* for complementation construction of P$_{pepV}$ (magenta highlight) and P$_{dat}$ (green highlight), respectively. (Overlapping sequences are underlined and bolded for differentiation). *pepV* and *dat* start codons are highlighted in gray. **(B)** Schematic representation of single-copy chromosomal complementation of *dat* from whole-genome-sequenced KPL4549 (*hla*::Tn (spc^R) *dat*::Tn (erm^R) *attB*::pLL39::P$_{dat}$*dat*). The Φ11 *attB* integration site is located 46 bp into the coding region of SAUSA300_RS10150. Recombination with the pLL39-derived complementation construct pAB004 (*P$_{dat}$dat*) occurred at 5′-CCATG|GGAAG-3′. The complementation construct (*P$_{dat}$dat*) was assembled at *SmaI* (5′-GGGCCC-3′) in the multiple cloning site (MCS) by Gibson assembly into pLL39 (see **Table S5** for complementation oligomers). Relevant plasmid elements include the spectinomycin resistance cassette (*SmR*), *Rep101* and SC101 origin in *E. coli*, and *rrnB* T1 transcriptional terminators to minimize transcriptional readthrough. The *P$_{dat}$dat* complementation insert is in the opposite orientation of the surrounding chromosomal genes, as shown on the displayed 5′ → 3′ strand.

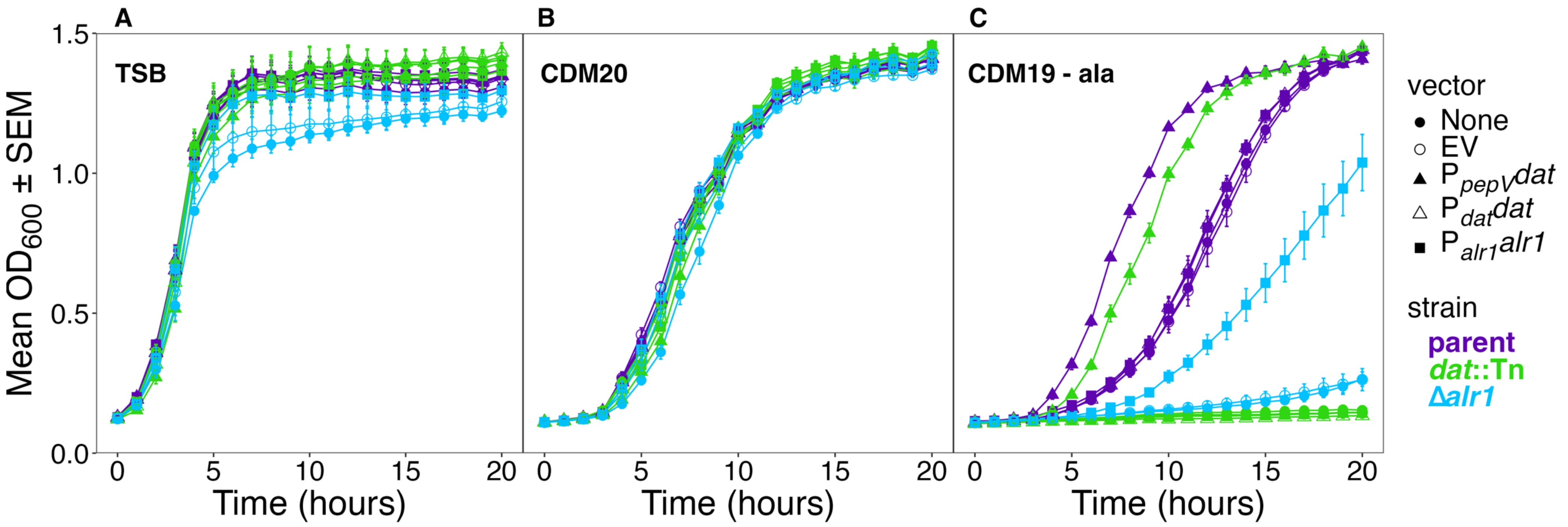

Figure S3. **Complete set of growth curves for all *S. aureus* strains (mutants and their complements) in the KPL4530 background.** Panels correspond to (**A**) TSB, (**B**) CDM20, and (**C**) CDM19-alanine liquid growth media. The parental *S. aureus* KPL4530 (JE2 *hla*::Tn(spc)) (purple) and isogenic *dat*::Tn(erm) (green) and Δ*alr1* (blue) mutants each carried either no vector (filled circles), empty vector (EV) pLL39 (empty circles), P_{pepV}*dat* (filled triangles), P_{dat}*dat* (empty triangles), or P_{alr1}*alr1* (filled squares), inserted at the Φ11 *attB* site. *n* = 3 independent experiments. Each data point is the average OD_{600} ± SEM of all 3 experiments at that time point. To assess differences in growth curves within each medium, we compared the area under the curve (AUC) using an LMM with strain as a fixed effect and experimental date as a random effect. We performed pairwise contrasts comparing the parental strain carrying either the empty vector (EV) or no vector (None) to all other strains. In addition, we evaluated contrasts among plasmid variants within each mutant background (*dat*::Tn and *Δalr1*). Selected comparisons were adjusted for multiple testing using the Holm method, with statistical significance defined as $p < 0.05$. Statistical analysis is in **Table S2B**.

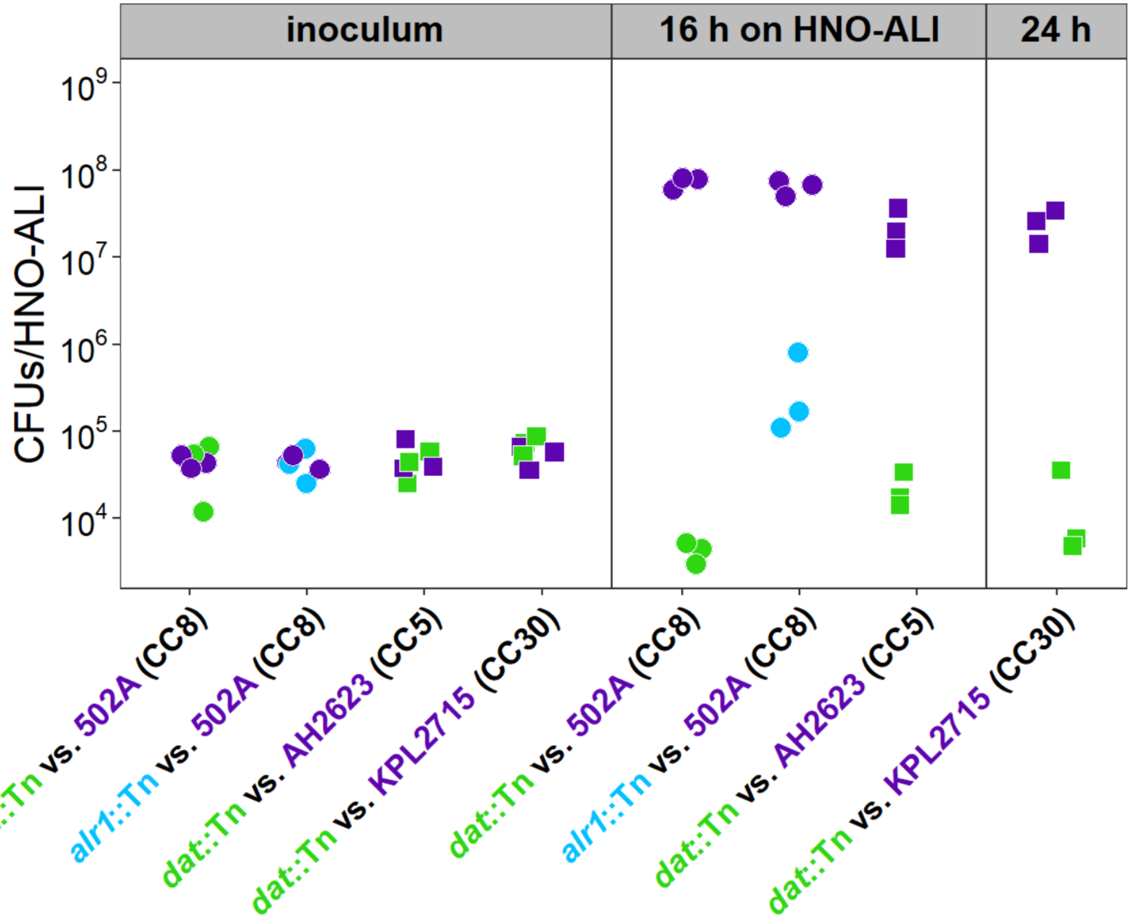

Figure S4. ***dat* contributes to *S. aureus* fitness during nasal colonization across multiple clonal complexes.** CFUs/HNO-ALI from *S. aureus* strains in Clonal Complex (CC) 8, 5, and 30. The parental strain (purple) outcompeted an isogenic mutant with a Tn disruption in either *dat* (green), or *alr1* (blue), respectively. HNO-ALI were cocolonized with 10^5 CFUS/HNO-ALI each of the parental *S. aureus* strains, (502A – CC8, AH2623 – CC5, and KPL2715 – CC30) and each mutant of interest for 16 or 24 h at 34 °C in n = 3 independent experiments in either of 2 HNO lines (HNO204, circles or HNO918, squares).

Table S3. P_{dat} and P_{pepV} region SAPPHIRE promoter prediction results

Strand	TSS position (bp relative to *pepV* start codon (ATG))	p-value	Predicted Promoter Sequence
**5' of *pepV* coding region**			
+	-574	0.005014	TAGTGTTTCAGGTTCTTTATCGTTAGTCA
+	-515	0.002222	ATGCGTGAAACTCATTCATAAGAAATACT
+	-300	0.000066	TTGTCTGTAGAATTTCTTTTCGAAATTCT
**+**	**-192**	**0.00424**	**TATCTCGAGCTCTTGAATATGTGTATTTT**
**+**	**-109**	**0.00287**	**AATAGCATCGTTTTGTTCCTTTTTATTAT**
**+**	**-76**	**0.002012**	**CTGACATCTTATTGTAGTTTTTATATCTT**
**+**	**-19**	**0.001817**	**TGTAGTTTTTATATCTTTTCATGTAAAAT**
**Within *pepV* coding region**			
+	149	0.000057	TGGTCCAGGTCCTCGTAAGGCATTAGACT
+	207	0.001138	TTTACAACACATGATGTGGATCATATTGC
+	438	0.004084	GTGGATTGGAAGAAACGTATTCATATGAT
+	487	0.001459	CTGATTGGAAATGTACGGATCGCTATTTT
+	544	0.004408	TTGCACCAGATGCAGAATTTCCATGTATT
+	660	0.001963	TTTAAATCTGGTGAACGTTACAACATGGT
+	788	0.003184	TAGTACTGTTGATAGTGGCATTCTAGTTT
+	863	0.003968	TGTGAATGCGGGTCTTTACTTACTAAAAT
+	872	0.003989	GGGTCTTTACTTACTAAAATTCTTAGCAT
**+**	**1210**	**0.002985**	**TTGTACAAAAGTTAGTTACTGCATATAGA**
**+**	**1420**	**0.000328**	**TTTATTCATTATGCGTGGAGGAATAATAT**

Table S4. Strains and plasmids used in this study.

Species	Strain	Int. Ref.	Characteristics	Reference
E. coli	NEB 5-alpha (#C2987)	KPL4489	High-efficiency DH5α-derived cloning strain (endA$^-$, T1 phage resistant) used for plasmid construction, Gibson assembly, and propagation	[1]
E. coli	NEB 5-alpha	KPL4565	NEB 5-αlpha transformed with Gibson assembled pKQ002 (Δ*alr1*)	This study
E. coli	NEB 5-alpha	KPL4534	NEB 5-αlpha transformed with Gibson assembled pAB001 (pLL39::P$_{alr1}$*alr1*)	This study
E. coli	NEB 5-alpha	KPL4535	NEB 5-αlpha transformed with Gibson assembled pAB004 (pLL39::P$_{dat}$*dat*)	This study
E. coli	NEB 5-alpha	KPL4536	NEB 5-αlpha transformed with Gibson assembled pAB009 (pLL39::P$_{pepV}$*dat*)	This study
E. coli	DC10b	KPL2100	dam$^+$ dcm$^-$ derivative of DH10B, intermediate cloning host used prior to transformation into *S. aureus*	[2]
E. coli	DC10b	KPL4566	DC10b (KPL2100) transformed with isolated pKQ002 (Δ*alr1*) from NEB 5-alpha (KPL4565)	This study
S. aureus	LAC	KPL4403	USA300 CA-MRSA strain LAC (MLST ST8, *spa* Type I, SCC*mec* Type IV, *agr* Type I)	[3]
S. aureus	LAC	KPL4447	USA300 MRSA LAC saturated transposon library, with 77,161 independent insertions in genome	[4]
S. aureus	JE2	KPL4465	*hla*::Tn *bursa aurealis* transposon mutant (SAUSA300_RS05720[1]) in JE2 from the Nebraska Transposon Mutant Library (NTML)	[5]
S. aureus	JE2	KPL4471	NTML *dat*::Tn *bursa aurealis* transposon mutant (SAUSA300_RS09265)	[5]
S. aureus	JE2	KPL4472	NTML *alr1*::Tn *bursa aurealis* transposon mutant (SAUSA300_RS11155)	[5]
S. aureus	JE2	KPL4474	NTML *fmtA*::Tn *bursa aurealis* transposon mutant (SAUSA300_RS05155)	[5]
S. aureus	RN4220	KPL4454	Restriction-deficient (*hsd*RMS$^-$) laboratory strain	[6]
S. aureus	RN4220	KPL4537	RN4220 transformed with pLL39 empty vector	This study
S. aureus	RN4220	KPL4538	RN4220 transformed with pAB004 (pLL39::P$_{dat}$*dat*) from NEB 5-alpha (KPL4535)	This study
S. aureus	RN4220	KPL4539	RN4220 transformed with pAB009 (pLL39::P$_{pepV}$*dat*) from NEB 5-alpha (KPL3536)	This study
S. aureus	RN4220	KPL4540	RN4220 transformed with pAB001 (pLL39::P$_{alr1}$*alr1*) from NEB 5-alpha (KPL4534)	This study
S. aureus	RN4220	KPL4542	***dat*::Tn (erm^R)**, transduced (Φ11) from NTML *dat*::Tn (SAUSA300_RS09265) into *S. aureus* RN4220	This study
S. aureus	RN4220	KPL4546	***alr1*::Tn (erm^R)** transduced (Φ11) from NTML *alr1*::Tn (SAUSA300_RS11155) into *S. aureus* RN4220	This study
S. aureus	JE2	KPL2115	Plasmid-free derivative of USA300 MRSA strain LAC	[5]
S. aureus	JE2	KPL4515	***dat*::Tn (erm^R)**, transduced (Φ11) from NTML *dat*::Tn (SAUSA300_RS09265) into *S. aureus* JE2	This study
S. aureus	JE2	KPL4516	***hla*::Tn (erm^R)**, transduced (Φ11) from NTML *hla*::Tn (SAUSA300_RS05720) (KPL4465) into JE2	This study
S. aureus	JE2	KPL4530	***hla*::Tn (spc^R)**, transduced (Φ11) from NTML *hla*::Tn (SAUSA300_RS05720) into JE2, followed by antibiotic cassette exchange via pSPC (erm^R → spc^R)	This study
S. aureus	JE2	KPL4532	**Δ*alr1*,** *S. aureus* JE2 transformed with isolated pKQ002 in *E. coli* DC10b (KPL4566) and allelic exchange performed for markerless deletion of *alr1* (See Methods)	This study
S. aureus	JE2	KPL4541	***hla*::Tn (spc^R) Δalr1**, transduced (Φ11) from *hla*::Tn (spc^R) into JE2 Δ*alr1* (KPL4532)	This study
S. aureus	JE2	KPL4545	***hla*::Tn (spc^R) *dat*::Tn (erm^R)**, transduced (Φ11) from *hla*::Tn (spc^R, KPL4530) into *dat*::Tn (erm^R, KPL4515)	This study
S. aureus	JE2	KPL4556	***hla*::Tn (spc^R) *fmtA*::Tn (erm^R)**, transduced (Φ11) from *fmtA*::Tn (erm^R, KPL4474) into *hla*::Tn (spc^R, KPL4530)	This study
S. aureus	JE2	KPL4553	***hla*::Tn (spc^R) attB::pLL39**, KPL4530 with the pLL39 empty vector integrated at the Φ11 *attB* site	This study
S. aureus	JE2	KPL4543	***hla*::Tn (spc^R) attB::pLL39::P$_{dat}$*dat***, KPL4530 with pAB004 (pLL39::P$_{dat}$*dat*) integrated at the Φ11 *attB* site	This study
S. aureus	JE2	KPL4552	***hla*::Tn (spc^R) attB::pLL39::P$_{pepV}$dat**, KPL4530 with pAB009 (pLL39::P$_{pepV}$*dat*) integrated at the Φ11 *attB* site	This study
S. aureus	JE2	KPL4544	***hla*::Tn (spc^R) attB::pLL39::P$_{alr1}$*alr1***, KPL4530 with pAB001 (pLL39::P$_{alr1}$*alr1*) integrated at the Φ11 *attB* site	This study
S. aureus	JE2	KPL4547	***hla*::Tn (spc^R) *dat*::Tn (erm^R) attB::pLL39**, KPL4545 with the pLL39 empty vector integrated at the Φ11 *attB* site	This study
S. aureus	JE2	KPL4549	***hla*::Tn (spc^R) *dat*::Tn (erm^R) attB::pLL39::P$_{dat}$*dat***, KPL4545 with pAB004 (pLL39::P$_{dat}$*dat*) integrated at the Φ11 *attB* site	This study
S. aureus	JE2	KPL4550	***hla*::Tn (spc^R) *dat*::Tn (erm^R) attB::pLL39::P$_{pepV}$*dat***, KPL4545 with pAB009 (pLL39::P$_{pepV}$*dat*) integrated at the Φ11 *attB* site	This study
S. aureus	JE2	KPL4548	***hla*::Tn (spc^R) *dat*::Tn (erm^R) attB::pLL39:: P$_{alr1}$*alr1*,** KPL4545 with pAB001 (pLL39::P$_{alr1}$*alr1*) integrated at the Φ11 *attB* site (extra copy of *alr1*)	This study
S. aureus	JE2	KPL4554	***hla*::Tn (spc^R) Δ*alr1* attB::pLL39**, *hla*::Tn (spc^R) Δ*alr1* (KPL4541) with pLL39 empty vector integrated at the Φ11 *attB* site	This study
S. aureus	JE2	KPL4551	***hla*::Tn (spc^R) Δ*alr1* attB::pLL39:: P$_{alr1}$*alr1***, KPL4541 with pAB001 (pLL39::P$_{alr1}$*alr1*) integrated at the Φ11 *attB* site	This study
S. aureus	JE2	KPL4555	***hla*::Tn (spc^R) *alr1*::Tn (erm^R),** transduced (Φ11) from NTML *alr1*::Tn (erm^R, KPL4472) into *hla*::Tn (spc^R, KPL4530)	This study
S. aureus	JE2	KPL4560	***dat*::Tn (tet^R)**, generated via antibiotic cassette exchange (erm^R → tet^R) from *dat*::Tn (erm^R, KPL4515)	This study
S. aureus	RN4220	KPL4561	***dat*::Tn (tet^R)**, transduced (Φ11) from *dat*::Tn (tet^R, KPL4560) into RN4220 (KPL4454)	This study
S. aureus	JE2	KPL4562	***dat*::Tn (tet^R) *hla*::Tn (spc^R)**, transduced (Φ11) from *hla*::Tn (spc^R, KPL4530) into *dat*::Tn (tet^R, KPL4560)	This study
S. aureus	502A	KPL4558	CC8 clinical isolate 502A once used for bacterial interference of invasive *S. aureus* infections (1963)	[7]
S. aureus	502A	KPL4567	***dat*::Tn (erm^R)**, transduced (Φ11) from *dat*::Tn (erm^R) in RN4220 (KPL4542) into *S. aureus* 502A (KPL4458)	This study
S. aureus	502A	KPL4567	***alr1*::Tn (erm^R)**, transduced (Φ11) from *alr1*::Tn (erm^R) in RN4220 (KPL4546) into *S. aureus* 502A (KPL4458)	This study
S. aureus	AH2623	KPL4559	Nasal Isolate susceptible to 80α phage, USA100 Blood Isolate #209	[8]
S. aureus	AH2623	KPL4563	***dat*::Tn (tet^R)**, transduced (Φ11) from *dat*::Tn (tet^R) (KPL4560) into *S. aureus* AH2623 (KPL4459)	This study
S. aureus	MNM000212	KPL2715	Primary nasal isolate (*mecA*+, CC30, ST30) per PubMLST	This study
S. aureus	KPL2715	KPL4360	Passage of KPL2715 sent for WGS (Microbial Genome Sequencing Center)	This study
S. aureus	KPL2715	KPL4564	***dat*::Tn (tet^R)**, transduced (Φ11) from *dat*::Tn (tet^R) (KPL4561) into *S. aureus* nasal isolate (KPL4360)	This study
Corynebacterium pseudodiphtheriticum	KPL1989	KPL1989	Primary adult human nostril isolate	[9]
**Plasmid**	**Host Strain**	**Int. Ref.**	**Characteristics**	**Reference**
pJB38	NE3001 (*E. coli* DH5α)		pUC19-based allelic exchange vector in *E. coli* and *S. aureus* (*Eco*[2] amp^R ; *Sau*[3] cam^R)	[10]
pSPC	NE3003 (*S. aureus* RN4220)	KPL4521	Allelic exchange plasmid containing homologous DNA from *bursa aurealis* with *aad9* (spectinomycin resistance) for exchange with *ermB* in *bursa aurealis* (*Eco* amp^R; *Sau* cam^R spc^R)	[10]
pTET	NE3005 (*S. aureus* RN4220)	KPL4522	Allelic exchange plasmid containing homologous DNA from *bursa aurealis* with *tetM* (tetracycline resistance) for exchange with *ermB* in *bursa aurealis* (*Eco* amp^R; *Sau* cam^R tet^R)	[10]
pLL39 (#15458, Addgene)	*E. coli*	KPL4528	*S. aureus-E. coli* shuttle vector (with tandem terminators at MCS) for single-copy integration at L54a *attB* and phi-11 *attB* sites (*Sau* spc^R)	[11]
pLL2787 (#15460, Addgene)	*E. coli*	KPL4529	Bacterial expression vector, encoding Φ11 integrase for single-copy integration at *attB* sites (*Eco* amp^R)	[11]
pKQ002	*S. aureus* JE2	KPL4532	pJB38-derived allelic exchange plasmid assembled by Gibson assembly containing ~700–900 bp flanking regions of *alr1* for markerless in-frame deletion in JE2 (*Eco* amp^R; *Sau* cam^R)	This study
pAB004	*S. aureus* RN4220	KPL4538	pLL39-derived vector for complementation, containing *dat* under its putative 5' intraoperon promoter (P$_{dat}$*dat*) (*Sau* spc^R)	This study
pAB009	*S. aureus* RN4220	KPL4539	pLL39-derived vector for complementation, containing *dat* under the *pepV* promoter (P$_{pepV}$*dat*) in operon (*Sau* spc^R)	This study
pAB001	*S. aureus* RN4220	KPL4540	pLL39-derived vector for complementation, containing *alr1* under its native promoter (P$_{alr1}$*alr1*) (*Sau* spc^R)	This study

bioRxiv preprint doi: https://doi.org/10.64898/2026.06.01.729472; this version posted [illegible]. The copyright holder for this preprint (which was not certified by peer review) is the author/funder, who has granted bioRxiv a license to display the preprint in perpetuity. It is made available under a CC-BY 4.0 International license.

[1] Locus tags correspond to *Staphylococcus aureus* gene annotations in AureoWiki.
[2] *Eco* = *E. coli*
[3] *Sau* = *S. aureus*

bioRxiv preprint doi: https://doi.org/10.64898/2026.06.01.729472; this version posted June 4, 2026. The copyright holder for this preprint (which was not certified by peer review) is the author/funder, who has granted bioRxiv a license to display the preprint in perpetuity. It is made available under a CC-BY 4.0 International license.

Table S5. Oligonucleotides used in this study.

Oligo #	Oligo Description	Sequence (5'-3')
**oKL926**	3' primer, 123 bp 3' of pJB38 MCS (multicloning site). Pairs with oKL927 for plasmid verification	CCCGAAAAGTGCCACCTGACGTC
**oKL927**	5' primer, 92 bp 5' of pJB38 MCS<br>Pairs with oKL926 for plasmid verification	CGAAAATGCCTCACATTTGTGCCACC
**oKL940**	5' primer, containing tail (underlined[4]) of pJB38 MCS. Paired with oKL941 yields 650 bp fragment 5' of *alr1* (SAUSA300_RS11155[5])	GAATTCGAGCTCGGTACCCGGATGAAAAGGTAATACCATATTTTCG
**oKL941**	3' primer, amplifies ~650 bp 5' of *alr1* (SAUSA300_RS11155), containing Gibson overlap (underlined) with 5' fragment downstream of *alr1*	GATAACAAATTTTATATGCACTGACCATAATTACTTCCTCCTGTAATTCCC
**oKL942**	5' primer, paired with oKL943 yields ~950 bp fragment 3' of *alr1* (SAUSA300_RS11155)	TATGGTCAGTGCATATAAAATTTGTTATC
**oKL943**	3' primer, containing tail (underlined) of pJB38 MCS, paired with oKL942 yields ~950 bp fragment 3' of *alr1* (SAUSA300_RS11155)	TCGACTCTAGAGGATCCCCCTTTAGGATGGAATACCTAAAGCC
**oKL944**	5' primer, paired with oKL945 amplifies *alr1* (SAUSA300_RS11155) and ~700-900 bp flanking regions	GCGCCAGTCTATCGATATAAGCACTG
**oKL945**	3' primer, paired with oKL944 amplifies *alr1* (SAUSA300_RS11155) and ~700-900 bp flanking regions	CCTCACTTCCCTTACAAAGCAAATGC
**oKL996**	3' primer in spc^R cassette to pair with primers located in genes of interest (oKL964, 965, 1056)	CCTAAAGAGCATGTAACTTTACTGG
**oKL997**	5' primer in spc^R cassette to pair with primer located in genes of interest (oKL964, 965, 1056)	GCCACCTGACGTCTAAGAAACC
**oKL1051**	5' primer in tet^R cassette to pair with primer located in genes of interest (oKL964, 965, 1056)	GGCTTAGTGTTTGTTTAGCAGAGC
**oKL1052**	3' primer in tet^R cassette to pair with primer located in genes of interest (oKL964, 965, 1056)	GCTTCCTAATTCTGTAATCGCTCC
**oKL938**	5' primer, paired with oKL939 amplifies *dat* (SAUSA300_S09265) and ~700 bp flanking regions	CTGATCATGCAGAAGCAAGAGTGC
**oKL939**	3' primer, paired with oKL938 amplifies *dat* (SAUSA300_S09265) and ~700 bp flanking regions	CAATACCTTCTGCTGATAATGCCGC
**oKL932**	5' primer, paired with oKL933 amplifies *hla* (SAUSA300_RS05720) and ~900 bp flanking regions	AATCTTGTACTGTTTGATATGGAACTCCTG
**oKL933**	3' primer, paired with oKL932, amplifies *hla* (SAUSA300_RS05720) and ~900 bp flanking regions	ATGAAGAAGTCCATACAAAATCCGCATC
**oKL184**	*Bursa aurealis* transposon-specific primer (Buster; minus orientation), 133 bp from Tn edge [5]	GCTTTTTCTAAATGTTTTTTAAGTAAATCAAGTAC
**oKL185**	*Bursa aurealis* transposon-specific primer (Upstream; plus orientation), 464 bp from Tn edge [5]	CTCGATTCTATTAACAAGGG
**oKL964**	3' gene-specific primer to verify presence of *bursa aurealis* insertion in *dat* (SAUSA300_S09265), amplifies 652 bp (oKL184) or 983 bp (oKL185)	CCAACTTTACCATCATTAAC
**oKL1056**	3' gene-specific primer to verify presence of *bursa aurealis* insertion in *alr1* (SAUSA300_RS11155), amplifies 948 bp (oKL184) or 1279 bp (oKL185)	CGTCTAATCATTCATTCG
**oKL965**	3' gene-specific primer to verify presence of *bursa aurealis* insertion in *hla* (SAUSA300_RS05720), amplifies 738 bp (oKL184) or 1069 bp (oKL185)	CTGAAGTTATCGGCTAAAG
**oKL1074**	3' gene-specific primer to verify presence of *bursa aurealis* insertion in *fmtA* (SAUSA300_RS05155), amplifies 934 bp (oKL184) or 1265 bp (oKL185)	GGCTTAGCATAGAAACCATATCG
**oKL1012**	5' complementation primer, with pLL39 3' MCS tail (underlined), 303 bp 5' of *alr1*, amplifies 1.5 kb with oKL1013	CGACTCTAGAGGATCCCCCACACATGAGCAACGTAAAATTG
**oKL1013**	3' complementation primer, with pLL39 5' MCS tail (underlined), 83 bp 3' of *alr1* stop codon, amplifies 1.5 kb with oKL1012	GAATTCGAGCTCGGTACCCTGAGAACCTCCAATTGATTCC
**oKL1014**	5' complementation primer, with pLL39 5' MCS tail (underlined), 252 bp 5' of *pepV*, amplifies 255 bp with oKL1015	CGACTCTAGAGGATCCCCGCATTTTCGTTCGGTTAATTACTG
**oKL1015**	3' complementation primer, with reverse complement of 5' *dat* coding region tail (underlined), amplifies 255 bp with oKL1014	CAAACTCACCATTTAAAAAAATTTTTTCCATTCGAAATCGACTTCCTTTTTTC
**oKL1016**	5' complementation primer, at 5' start of *dat* coding region, amplifies 951 bp with oKL1017	GAAAAAATTTTTTTAAATGGTGAGTTTG
**oKL1017**	3' complementation primer, with pLL39 5' MCS tail (underlined), 105 bp 3' of *dat* stop codon, amplifies 951 bp (oKL1017) or1874 bp (oKL1018)	GAATTCGAGCTCGGTACCCGTTTTCTAAACAACACTTTTAAGC
**oKL1018**	5' complementation primer, with pLL39 5' MCS tail (underlined), 371 bp 5' of dat coding region, amplifies 1874 bp with oKL1017	CGACTCTAGAGGATCCCCACTTACGCTACCCAGAAGG
**oKL1039**	5' primer, 412 bp 5' of pLL39 *att* site	GTTAGAGAATGACCGGTTTACC
**oKL1040**	3' primer, 333 bp 3' of pLL39 *att* site	CGTGCATAATAAGCCCTACAC
**oKL1041**	5' primer 829 bp 5' of JE2 *attB* insertion site, to pair with pLL39 oligos	GCCATATCGACCTTCTGTATC
**oKL1042**	3' primer 820 bp 3' of JE2 *attB* insertion site, to pair with pLL39 oligos	CGGGTCATTTCTAGTCGTTAC

[4] Underline indicates overlap region for Gibson assembly
[5] Locus tags correspond to *Staphylococcus aureus* gene annotations from AureoWiki

REFERENCES

1. Anton BP, Raleigh EA. Complete Genome Sequence of NEB 5-alpha, a Derivative of Escherichia coli K-12 DH5alpha. Genome Announc. 2016;4(6). Epub 20161110. doi: 10.1128/genomeA.01245-16. PubMed PMID: 27834703; PubMed Central PMCID: PMCPMC5105096.
2. Monk IR, Shah IM, Xu M, Tan MW, Foster TJ. Transforming the untransformable: application of direct transformation to manipulate genetically Staphylococcus aureus and Staphylococcus epidermidis. mBio. 2012;3(2). Epub 20120320. doi: 10.1128/mBio.00277-11. PubMed PMID: 22434850; PubMed Central PMCID: PMCPMC3312211.
3. Miller LG, Perdreau-Remington F, Rieg G, Mehdi S, Perlroth J, Bayer AS, et al. Necrotizing fasciitis caused by community-associated methicillin-resistant Staphylococcus aureus in Los Angeles. N Engl J Med. 2005;352(14):1445-53. PubMed PMID: 15814880.
4. Grosser MR, Paluscio E, Thurlow LR, Dillon MM, Cooper VS, Kawula TH, et al. Genetic requirements for Staphylococcus aureus nitric oxide resistance and virulence. PLoS Pathog. 2018;14(3):e1006907. Epub 2018/03/20. doi: 10.1371/journal.ppat.1006907. PubMed PMID: 29554137; PubMed Central PMCID: PMCPMC5884563.
5. Fey PD, Endres JL, Yajjala VK, Widhelm TJ, Boissy RJ, Bose JL, et al. A genetic resource for rapid and comprehensive phenotype screening of nonessential *Staphylococcus aureus* genes. mBio. 2013;4(1):e00537-12. Epub 2013/02/14. doi: 10.1128/mBio.00537-12. PubMed PMID: 23404398; PubMed Central PMCID: PMCPMC3573662.
6. Kreiswirth BN, Lofdahl S, Betley MJ, O'Reilly M, Schlievert PM, Bergdoll MS, et al. The toxic shock syndrome exotoxin structural gene is not detectably transmitted by a prophage. Nature. 1983;305(5936):709-12. doi: 10.1038/305709a0. PubMed PMID: 6226876.
7. Shinefield HR, Ribble JC, Boris M, Eichenwald HF. Bacterial interference: its effect on nursery-acquired infection with Staphylococcus aureus. I. Preliminary observations on artificial colonzation of newborns. Am J Dis Child. 1963;105:646-54. PubMed PMID: 13977323.
8. Mootz JM, Benson MA, Heim CE, Crosby HA, Kavanaugh JS, Dunman PM, et al. Rot is a key regulator of Staphylococcus aureus biofilm formation. Molecular microbiology. 2015;96(2):388-404. Epub 20150226. doi: 10.1111/mmi.12943. PubMed PMID: 25612137; PubMed Central PMCID: PMCPMC4467170.
9. Brugger SD, Eslami SM, Pettigrew MM, Escapa IF, Henke MT, Kong Y, et al. *Dolosigranulum pigrum* Cooperation and Competition in Human Nasal Microbiota. mSphere. 2020;5(5):e00852-20. Epub 2020/09/11. doi: 10.1128/mSphere.00852-20. PubMed PMID: 32907957; PubMed Central PMCID: PMCPMC7485692.
10. Bose JL, Fey PD, Bayles KW. Genetic tools to enhance the study of gene function and regulation in Staphylococcus aureus. Appl Environ Microbiol. 2013;79(7):2218-24. Epub 2013/01/29. doi: 10.1128/AEM.00136-13

AEM.00136-13 [pii]. PubMed PMID: 23354696; PubMed Central PMCID: PMC3623228.
11. Luong TT, Lee CY. Improved single-copy integration vectors for Staphylococcus aureus. J Microbiol Methods. 2007;70(1):186-90. Epub 20070424. doi: 10.1016/j.mimet.2007.04.007. PubMed PMID: 17512993; PubMed Central PMCID: PMCPMC2001203.
